# Modelling the effects of antimicrobial metaphylaxis and pen size on bovine respiratory disease in high and low risk fattening cattle

**DOI:** 10.1186/s13567-022-01094-1

**Published:** 2022-10-04

**Authors:** Sébastien Picault, Pauline Ezanno, Kristen Smith, David Amrine, Brad White, Sébastien Assié

**Affiliations:** 1 INRAE, Oniris, BIOEPAR, 44300 Nantes, France; 2 Beef Cattle Institute, Kansas State University, Manhattan, KS 66506 USA

**Keywords:** Epidemiological modelling, bovine respiratory disease, antimicrobial usage, disease control, farming practices, stochastic models

## Abstract

Bovine respiratory disease (BRD) dramatically affects young calves, especially in fattening facilities, and is difficult to understand, anticipate and control due to the multiplicity of factors involved in the onset and impact of this disease. In this study we aimed to compare the impact of farming practices on BRD severity and on antimicrobial usage. We designed a stochastic individual-based mechanistic BRD model which incorporates not only the infectious process, but also clinical signs, detection methods and treatment protocols. We investigated twelve contrasted scenarios which reflect farming practices in various fattening systems, based on pen sizes, risk level, and individual treatment vs. collective treatment (metaphylaxis) before or during fattening. We calibrated model parameters from existing observation data or literature and compared scenario outputs regarding disease dynamics, severity and mortality. The comparison of the trade-off between cumulative BRD duration and number of antimicrobial doses highlighted the added value of risk reduction at pen formation even in small pens, and acknowledges the interest of collective treatments for high-risk pens, with a better efficacy of treatments triggered during fattening based on the number of detected cases.

**Supplementary Information:**

The online version contains supplementary material available at 10.1186/s13567-022-01094-1.

## Introduction

Bovine respiratory disease (BRD) is a multi-pathogen disease, caused by bacteria (e.g., *Mycoplasma bovis*, *Mannheimia haemolytica*) and viruses (e.g., the respiratory syncytial bovine virus, the bovine herpesvirus type 1, or the bovine viral diarrhoea virus) [1–3]. BRD is a major burden in fattening farms, as a large proportion of young beef calves develop BRD short after pen formation [4]. Transportation and mixing associated with pen formation as well as the diversity of pathogens involved in BRD make this disease difficult to anticipate and control, leading to a broad use of antimicrobials to limit the impact of BRD on animal health and welfare, as well as economic losses due to reduced weight gain [4]. Most models developed so far focused on identifying risk factors and statistical predictors for BRD occurrence and impact [5, 6]. Mechanistic models, where all processes involved in the dynamics of the pathosystem are made explicit, are a complementary lever to gain insights on epidemiological issues, compare realistic control measures at individual or collective scale, and identify possible trade-offs between health, welfare, or economic decision criteria [7]. Mechanistic modelling is thus an effective way to explore the best BRD control strategies to implement on-farm.

In a preliminary study [8], a stochastic mechanistic BRD model was designed to represent fattening pens in French farms, accounting for the variability of host response to infection. To keep the model simple and cope with the lack of knowledge and data regarding the interactions between the multiple pathogens involved in BRD, we assumed an “average” pathogen. This first model made it possible to compare strategies based on the combination of a treatment protocol (either an individual or a collective treatment) and a detection method (either by visual appraisal only, or with an additional screening based on manual temperature measurement 12 h after the first case which reflects classical French veterinary recommendations, or with sensor-based temperature measurement).

Yet, this model targeted typical European farms with small pen sizes (10 animals). To expand the scope of this study, increase the robustness of the underlying assumptions and the confidence in the outcomes, the model was substantially revised to account for contrasted farming practices regarding pen sizes, possible collective treatments at pen formation or during fattening, or the assignment of a risk level to pens. This new study was designed to represent fattening facilities receiving weaned calves (between 200 and 320 kg) sold by suckler herds, then fed either in large outdoor feedlots or in smaller indoor barns, which can represent typical situations in many countries.

To allow for a more realistic account of BRD detection, a finer-grained and explicit description of clinical states was designed. The severity of BRD indeed depends both on the nature of clinical signs (from nasal discharge or coughing to anorexia or depression) and on their intensity, which led us to consider explicit mild and severe cases, and to include a small, but not negligible, proportion of deaths [9]. Also, undetected BRD episodes are responsible for a reduction in the average daily gain during fattening [10], which highlights the need for a detailed assessment of detection methods and subsequent measures.

Yet, reducing BRD severity must involve a balance between reducing disease duration at pen scale and the amount of antimicrobials required to do so. The aim of this study was thus to compare the impact of various farming and health practices in terms of antimicrobial usage (AMU) and reduction of BRD severity, in the perspective of an individualized medicine and of a reasoned usage of antimicrobials.

## Materials and methods

### BRD model: assumptions and processes

Our work aims at supporting individualized veterinary medicine. Hence, the model designed for BRD was individual-based (to ensure a fine-grained detail level), stochastic (to account for intrinsic variability in biological processes and observation), and mechanistic (to represent explicit processes and identify levers in disease control), with discrete time steps of 12 h which corresponds to the delay between consecutive visual appraisals of beef cattle at feeding. Discussing, implementing and assessing new assumptions was facilitated by the EMULSION platform [11] (version 1.2), which makes it possible to describe all model components (structure, processes, parameters) as a human-readable, flexible and modular structured text file, processed by a generic simulation engine, hence enabling modellers, computer- and vet scientists to co-design or revise the model at any moment (the whole EMULSION model, including parameters, is available on a public Git repository and in Additional file 1: section 2).

**Figure 1:**
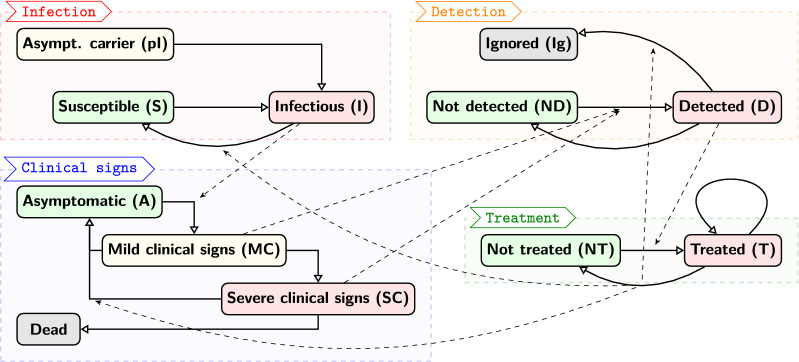
**Overview of the mechanistic BRD model.** The model incorporated four processes (infection, clinical signs, detection, treatment) associated to individual states (rounded boxes), which could evolve by themselves (plain arrows) but also influenced each other (dashed arrows). For instance, animals becoming infectious (I) also started expressing mild clinical signs (MC), which could evolve towards severe clinical signs (SC). Both could be detected (D), which led to a first treatment (T) that could be repeated. When the treatment was successful, it made the animal return to susceptible (S) and asymptomatic (A) states. If successive treatments failed, it was stopped and the animal was no longer considered for further treatments, thus markes as “ignored” (Ig).

An earlier mechanistic, stochastic, individual-based model of BRD [8] which had been developed in the context of typical French fattening farms, was adapted to encompass a broader range of farming practices and to more accurately reflect the onset of clinical signs and their detection. The new model integrated four explicit processes: the infectious process, the onset of clinical signs, the detection of BRD cases and the treatment of animals. Figure 1 provides an overview of these four processes and their interactions (full details on each process are available in Additional files 1A–E).

The first process (Additional files 1A, B) represented the evolution of health states. In our preliminary study [8] the infectious model was a SIS model where susceptible animals (S) could become infectious (I), then returned to S and so on, assuming a frequency-dependent force of infection. However, BRD results not only from transmission between animals [12] but also from asymptomatic pathogen carriage: pathogens can be already present in young beef calves and trigger a BRD episode after stress events [13, 14], especially in the first few weeks consecutive to pen formation [4]. Thus, we introduced a pre-infectious stage (pI) to represent asymptomatic carrier calves which eventually developed BRD after a delay. Animals in pI state could also become infectious in the meanwhile, due to contacts with other infectious individuals. When infection ended, animals returned to susceptible, assuming they could be re-infected by the “average” pathogen, as animals could be infected by several different pathogens (Additional files 1A, B). We assumed no between-pen transmission, as this situation is not documented enough to provide suitable transmission estimates. Besides, in small-pen fattening facilities, few calves arrive at the same time: as BRD mostly occur short after pen formation [4], such successive arrival of small groups of animals limits the risk that a new pen will be exposed to BRD episodes occurring at the same time in other pens.

The second process (Additional file 1C) represented the explicit onset of clinical signs, assuming four main stages. Susceptible individuals were asymptomatic (A); when becoming infectious, they started expressing mild clinical signs (MC). Then, after a delay, they could either develop severe clinical signs (SC) with probability p_severe_clinical, or return to A when infection was over. Finally, a few animals in SC state could die from BRD with probability p_death, but most of them returned to A when infection was over. This mortality was neglected in the preliminary study [8], but could not be ignored anymore in large pen sizes [9].

The third process (Additional file 1D), detection, relied on visual appraisal of clinical illness at feeding time, i.e. every 12 h. Visual observations of clinical illness are known to have a low sensitivity, which varies a lot depending on studies, from 0.27 (with 95% credible interval 0.12–0.65) [15] to 0.618 (97.5% CI 0.557–0.684) [16]. Assuming the sensitivity depended on the severity of clinical signs, we used Se_MC = 0.3 and Se_SC = 0.6 for MC and SC, respectively. Also, as BRD is by far the most prevalent disease in the beginning of feeding, we assumed a high specificity (Sp = 0.9) consistently with [15]. Finally, as detection could occur in the mechanistic model at any time step while animals were asymptomatic (false positives) or affected by mild or severe clinical signs respectively, we had to transform the specificity and the sensitivities into detection probabilities (respectively p_det_A, p_det_MC, p_det_SC) per time unit (i.e. per hour). These probabilities were calculated as expressed in Equations (1)–(3), based on the expected value of the durations in A, MC and SC states. Average durations in MC and SC were based on the distributions used in the model for mild clinical signs and for the infectious state (Additional file 1: “Complete EMULSION BRD model” section), whereas dur_A_expected was set to the total fattening duration. These three probabilities are then used in the transitions from ND to D states (Additional file 1D).

1 $$p\_\det \_A = 1 - Sp^{{\frac{1}{dur\_A\_expected}}}$$

2 $$p\_\det \_MC = 1 - \left( {1 - Se_{MC} } \right)^{{\frac{1}{dur\_MC\_expected}}}$$

3 $$p\_\det \_SC = 1 - \left( {1 - Se_{SC} } \right)^{{\frac{1}{dur\_SC\_expected}}}$$

The fourth process (Additional file 1E) represented an “average” antimicrobial treatment protocol during the fattening period. Any detected individual receives an individual treatment, consisting of administering up to three consecutive doses of antimicrobials. Each animal receiving an antimicrobial dose performed a random trial (with probability p_success = 0.8) to determine the outcome of the treatment. The effect of the treatment was considered after a period of 48 h. After that delay, animals could already be free of clinical signs due to the intrinsic dynamics of the infectious process, or still clinical with a treatment considered successful: in both cases, they returned to the non-treated state. On the contrary, animals with a negative outcome and still clinical signs were treated again. Animals with clinical signs 48 h after receiving a third antimicrobial doses were not considered for further treatments (marked through detection status “Ignored”). In addition to this individual treatment, we also considered the possibility to trigger a collective treatment at pen scale when the cumulate incidence reached a fixed threshold (depending on pen size). In that case, all animals not already under treatment in the pen received an antimicrobial dose, then they were followed individually according to the rules described above. In the treatment protocol, we did not explicitly consider the case of an infection caused only by a virus, for four reasons. First, viruses involved in BRD are rarely found without bacterial co-infection [17, 18]; second, when cases occur, most often no analyses are performed to identify the nature of the pathogen agent; third, detected animals are treated with antimicrobials anyway to prevent bacterial superinfections; and fourth, the stochasticity in treatment success and the possibility for animals to recover within each 48 h period could also represent what would happen with viral infections.

### Scenarios

We envisaged twelve scenarios resulting from the combination of two pen sizes (S: small, vs. L: large), three risk levels at pen formation (LR: low risk with a small proportion of pI animals; HR: high risk with a higher proportion; HRA: high risk mitigated by antibioprevention), and two treatment strategies (I: individual treatment only; C: also allowing collective treatment during fattening). Parameter values characterising the twelve scenarios are summarized in Table 1.

**Table 1:** **Parameters used to characterize scenarios depending on pen size and risk level**

Parameter name	Role	Small			Large		
		LR	HRA	HR	LR	HRA	HR
pen_size	Size of each pen	10 [1]			100 [16]		
metaphylaxis_threshold (best guess)	Proportion of cumulate detected cases with severe clinical signs above which collective treatment is triggered	0.4			0.15		
use_antibioprevention	Use long-acting antimicrobials just before fattening	0	1	0	0	1	0
initial_prevalence (best guess)	Proportion of asymptomatic carriers (pI) at the beginning of fattening	0.1	0.15	0.3	0.02	0.1	0.2

Two pen sizes (“large” and “small”) and two risk levels (LR, low risk and HR, high risk) were considered. In addition, we also considered high risk pens with antibioprevention (HRA), i.e. where a collective treatment with long-acting antimicrobials is given to all animals before fattening, assuming a reduction of half of the initial prevalence.

The first major difference in fattening practices is pen size indeed, which can vary by an order of magnitude, for instance from small pens of 10 animals, often fed indoor, as in France [1] to larger pens of 100 animals in the open air as in the US [16].

In addition, pens can be classified as “low-risk” or “high-risk” regarding BRD occurrence, depending on multiple factors, especially the diversity of origins and distance travelled by animals [9, 14, 19, 20]. Such a prior assessment of the risk level of animals on arrival has been widely studied and is quite common in large-pen farms, whereas in small-pen systems, risk assessment is still an applied research question [21]. However, we assumed that we could consider similar conditions for large- and small-pen systems. Also, antibioprevention (also called “metaphylaxis at arrival” or “preventive metaphylaxis”) is a common practice to mitigate the risk in high-risk pens [22, 23], by treating all animals with long-acting antimicrobials before fattening starts. Antibioprevention reduces BRD prevalence by a factor that varies a lot depending on the nature and possible interactions between drugs [24], and delays in the onset of clinical cases [25]. Consistently, we assumed that antibioprevention reduced the initial prevalence in high-risk pens by half, and added 2 weeks on average to the delay before pI animals became infectious. This delay was modelled explicitly by adding a random duration to the original distribution in pI state when the scenario specified the use of antibioprevention.

Finally, a collective treatment (also called “curative metaphylaxis”) can also be set up when several cases are detected [25], to reduce the prevalence and avoid transmission by undetected animals. We assumed that the threshold used to trigger a collective treatment was based on the cumulative proportion of detected cases and that this proportion was indeed not the same in small and large pens.

However, we assumed that animals were exposed to similar pathogens, i.e. that we could use the same characteristics (transmission rate, infection duration, morbidity and lethality) for the average pathogen in all scenarios. Most model parameters were set according to existing experimental data [12, 26] or, if not available, assumed within the range of the best estimates.

### Model outputs

The individual-based model made it possible to track individual events and to aggregate them at pen scale. For each stochastic replicate we recorded the number over time of animals that were infectious, with severe clinical signs, detected (with the detection dates) and treated. We recorded outputs after 100 days on feed: the number of distinct animals in each state, the associated cumulative durations (at pen scale, i.e. in animals × days), the date and amplitude of the epidemic peak (maximum number of detected cases), the number of deaths, and the total number of antimicrobial doses used during the period. We also traced how many detections occurred or failed (i.e. the number of animals detected while being in states A, MC and SC respectively as well as the number of animals that stayed undetected while being in those states), to calculate the proportion of detections by mild or severe clinical signs and the proportion of false positives in the simulation. We ran 500 stochastic replicates of large-pen (100 animals) scenarios and 5000 replicates of small-pen ones (10 animals); then, for each small-pen scenario, we randomly constructed groups of 10 pens to aggregate some simulation results (cumulative durations, number of deaths, amount of antimicrobials), to represent 500 farms that would have fattened 100 animals, hence making large- and small-pen scenarios comparable on those outputs.

These simulation outputs were used to analyse the dynamics of BRD cases simulated to ensure that they were consistent with data from previous studies or from the literature, but also to calculate the effectiveness of BRD control based on the trade-off between the cumulative duration of BRD impact at pen scale (assuming severe clinical signs as a good proxy for the disease severity) and the total number of antimicrobial doses used to reach that result.

### Sensitivity analysis

To better characterize the behaviour of the model and the impact of parameter uncertainty, we carried out a sensitivity analysis for six of the twelve scenarios that we considered an acceptable compromise between antimicrobial usage and disease control in an antibioreduction perspective, i.e. relying on individual treatments only when the risk level is low (LR) or was already reduced by antibioprevention (HRA), and enabling collective treatments in high-risk scenarios (HR). The sensitivity analysis incorporated 11 major model parameters (Table 2), involved in the processes describing infection, detection and clinical signs onset. Each model parameter was used at its nominal value and with a variation of ± 20% (except for the specificity that cannot exceed 1). The corresponding experiment plan was based on a fractional factorial design incorporating first-order parameter interactions and generated using the R library “planor” [27] (hence, reducing the 3^11^ possible combinations of parameter values to 3^5^ = 243 parameter settings per scenario). For each parameter setting, we carried out 500 stochastic replicates for large-pen scenarios and 5000 for small-pen ones. The sensitivity analysis targeted 6 model outputs: the date of the first detected BRD base, the day and height of the detection peak, the number of deaths, the total number of antimicrobial doses, and, for scenarios enabling collective treatments, the proportion of pens where a collective treatment was triggered (Table 3). For each scenario, an analysis of variance (ANOVA) was performed to identify the sensitivity index, i.e. the contribution of model parameters to the variance of the outputs: for each output, a linear regression model was fitted with the principal effects of the parameters and their first-order interactions. The contribution of parameter *p* to the variation of output *o* is calculated as described in Equation 4, where $SS_{tot}^{o}$, $SS_{p}^{o}$ and $SS_{p:p^{\prime}}^{o}$ are, respectively, the total sum of squares of the model, the sum of squares related to the principal effect of parameter *p*, and the sum of squares related to the interaction between parameters *p* and *p′*, for output *o*.

4 $$C_{p}^{o} = \frac{{SS_{p}^{o} + \frac{1}{2}\mathop \sum \nolimits_{p^{\prime}} SS_{p:p^{\prime}}^{o} }}{{SS_{tot}^{o} }}$$

**Table 2:** **Nominal values of parameters included in the sensitivity analysis**

Parameter name	Role	Nominal value	Source
mean_dur_I	Mean duration in I state (h)	5 * 24	Assumed
mean_dur_pI	Mean duration in pI state (h)	3 * 24	Assumed
pathogen_transmission_rate	Transmission rate by infectious individuals (h^−1^)	0.01	Assumed
p_death	Probability that an animal with severe clinical signs eventually dies from BRD	0.05	Best guess
dur_MC_fact	Factor modifying the delay between infection and onset of severe clinical signs	1	
p_severe_clinical	Probability that an animal with mild clinical signs eventually develops severe clinical signs	0.5	Best guess
dur_before_death	Duration of severe clinical signs before death for animals dying from BRD (h)	10 * 24	Assumed
Se_MC	Sensitivity of the detection based on mild clinical signs	0.3	[15]
Se_SC	Sensitivity of the detection based on severe clinical signs	0.6	[16]
Sp	Specificity of the detection based on clinical signs	0.9	[15]
p_recovery	Probability that one antimicrobial dose will stop the infection	0.8	Best guess

**Table 3:** **Model outputs considered in the sensitivity analysis**

Output name	Description
% collective	Proportion of pens where a collective treatment was triggered during the fattening period
First detection	Date of first BRD case detection
Nb deaths	Number of deaths during the whole fattening period, per 100 animals
Nb doses	Number of antimicrobial doses used during the whole period, per 100 animals
Peak day	Date of the peak of severe clinical cases during the whole period
Peak height	Maximum number of animals with severe clinical cases at the same time during the whole period

Outputs values are considered for a period of 100 days on feed and 500 stochasticreplicated for large = pen scenarios, 5000 for small-pen ones.

## Results

### BRD dynamics

**Figure 2:**
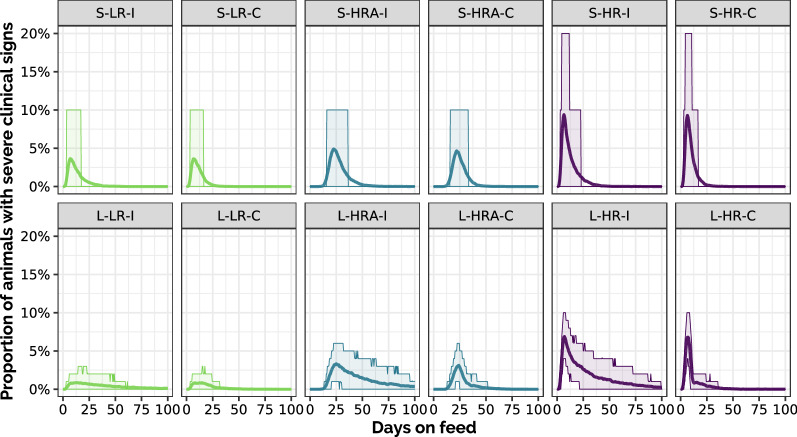
**Temporal dynamics of the occurrence of severe clinical signs.** Proportion of animals with severe clinical signs over time in each scenario: mean value (line) and 10^th^–90^th^ percentiles (ribbon) calculated on 500 stochastic replicates for large pens, 5000 for small pens. First row: small-pen scenarios (S), second row: large-pen scenarios (L); green: low risk (LR), blue: high risk with antibioprevention (HRA), purple: high risk (HR); for each color: individual treatment only (I) on the left, with collective treatment enabled during fattening (C) on the right.

Figure 2 presents the temporal dynamics for the occurrence of severe clinical signs at pen scale, on average and 10^th^–90^th^ percentile for the twelve scenarios (on 500 replicates for large-pen scenarios and 5000 for small-pen ones). As expected, the occurrence of severe clinical signs was mainly driven by the risk level. The use of collective treatment during fattening reduced the period during which severe clinical signs occurred, rather than of the amplitude of the episodes. Also, compared to high-risk (HR) scenarios, the use of antibioprevention (HRA) reduced and delayed the peak of cases with severe clinical signs.

**Figure 3:**
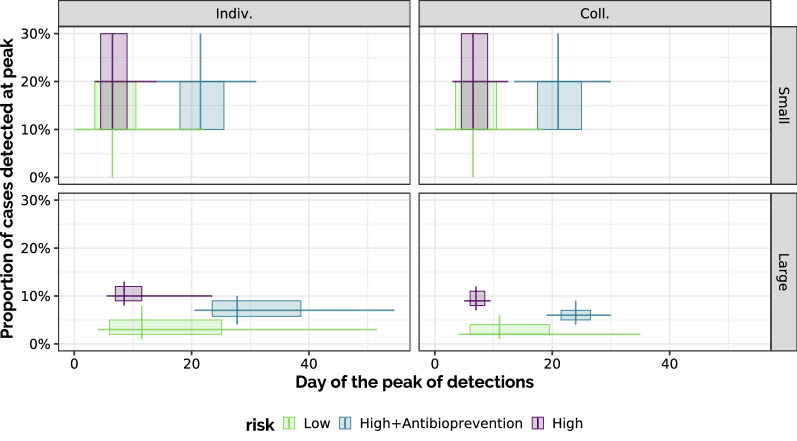
**Dates and amplitudes of detection peaks.** Each detection peak is characterised by the maximum number (y-axis) of animals detected over time in a stochastic replicate and by the date at which this maximum was reached (x-axis). Boxes extend from 1^st^ to 3^rd^ quartiles in each axis, lines are positioned at the median and extend from 10 to 90^th^ percentiles. 500 (resp. 5000) stochastic replicates were conducted in large-pen (resp. small-pen, first row) scenarios (second row). First column: individual treatment only (I); second column: with collective treatment enabled during fattening (C).

**Figure 4:**
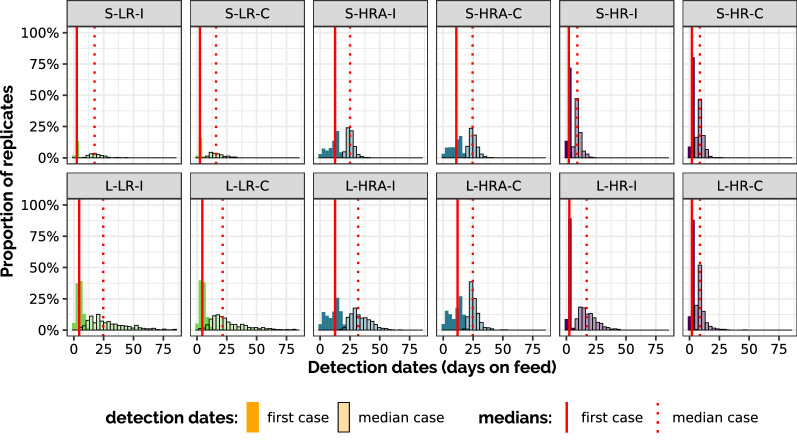
**Distributions of detection dates.** Histogram representing how the dates of first detection (“first case”, dark) and the median date of detection in each stochastic replicate (“median case”, light) are distributed over the set of stochastic replications. Vertical red lines represent the medians of the first (solid line) and median (dashed line) detection dates over the set of stochastic repetitions. First row: small-pen scenarios (S), second row: large-pen scenarios (L); green: low risk (LR), blue: high risk with antibioprevention (HRA), purple: high risk (HR); for each color: individual treatment only (I) on the left, with collective treatment enabled during fattening (C) on the right.

The date and amplitude of detected epidemic peaks, but also their variability, were also examined for all scenarios (Figure 3), especially showing that the use of a collective treatment during fattening in large-pen high-risk scenarios (with or without antibioprevention) induced a substantial reduction in peak date variability. Figure 4 presents representing how the dates of first detection and the median date of detection in each stochastic replicate are distributed over the set of stochastic replications in each scenario. The distributions of detection peaks and detection dates appeared consistent with observations reporting that most BRD cases usually occur during the first 45 days of the fattening period [16].

### BRD detection

**Figure 5:**
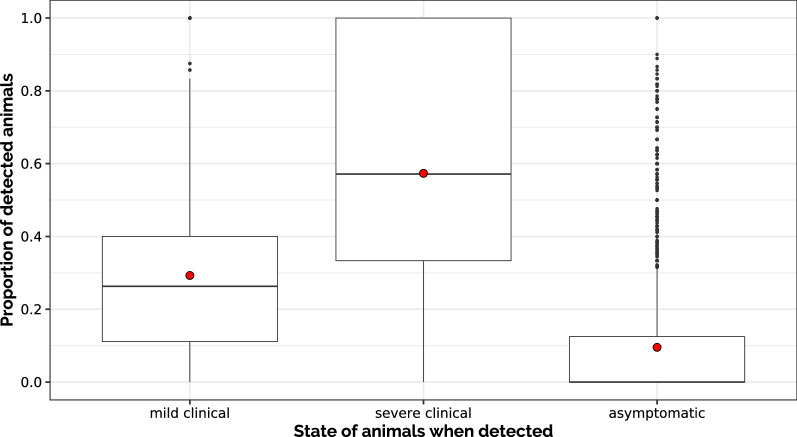
**Proportion of detections in each clinical state.** Distribution (boxplots) and average values (red dots) of the proportion of animals with mild clinical states (respectively, with severe clinical signs and asymptomatic) that were detected (aggregated over all scenarios).

On average, about 30% (respectively, 60%) of animals were detected during the period in which they expressed mild (resp., severe) clinical signs (Figure 5), which is in line with the values of sensitivity parameters (Se_MC = 0.3 and Se_SC = 0.6) used to calculate p_det_MC and p_det_SC (the detection probabilities for animals in MC or SC states during one time unit). On average, 10% of animals were considered detected though asymptomatic (Figure 5), which is also consistent with the value of the specificity parameter (Sp = 0.9) used to calculate p_det_A.

### BRD burden and effectiveness of control strategies

**Figure 6:**
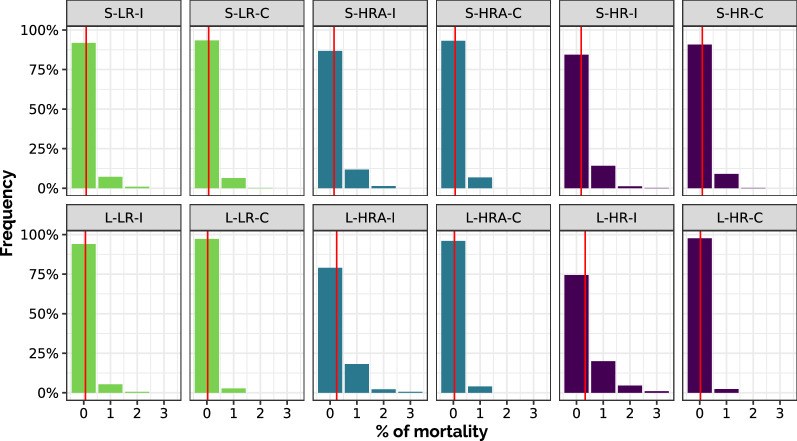
**Mortality.** Distribution of the number of dead animals cumulated over 100 days per 100 animals (vertical red line: mean) in the 12 scenarios (calculated on 500 stochastic replicates for large-pen scenarios, 5000 replicates aggregated 10 by 10 for small-pen scenarios). First row: small-pen scenarios (S), second row: large-pen scenarios (L); green: low risk (LR), blue: high risk with antibioprevention (HRA), purple: high risk (HR); for each color: individual treatment only (I) on the left, with collective treatment enabled during fattening (C) on the right.

The first indicator considered for BRD impact was the mortality (Figure 6), which as expected increased with the risk level. In low-risk scenarios, the death of one calf over 100 was rare (less than 10% in 500 stochastic replicates) and this was also the case in all small-pen scenarios (with a maximum of about 15% of stochastic replicates with one death). In large-pen scenarios, the situation rapidly deteriorated with the risk level when individual treatments only were allowed, with about 80% of stochastic replicates experiencing at least one dead animal in HRA, and about 70% in HR. However, the possibility of triggering a collective treatment proved effective in reducing mortality to the same level as in the low-risk situation.

**Figure 7:**
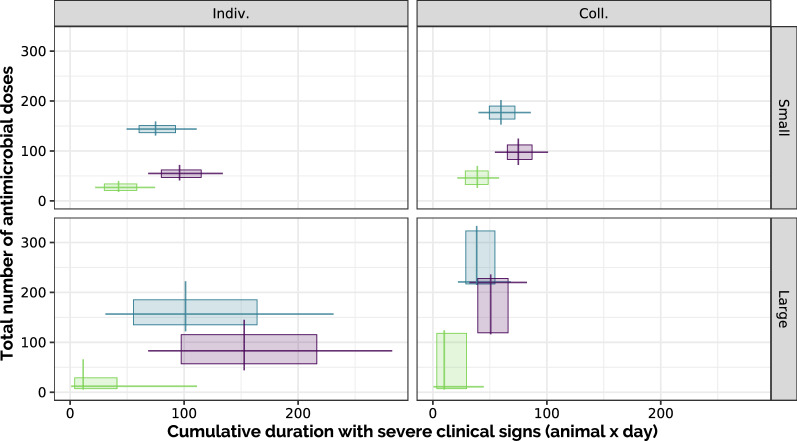
**Antimicrobial usage vs. disease duration per 100 animals.** Total amount of antibiotics doses required in each scenario to fatten 100 young beef bulls for 100 days, compared to the cumulative duration of severe clinical signs. Boxes extend from 1^st^ to 3^rd^ quartiles in each axis, lines are positioned at the median and extend from 10 to 90^th^ percentiles. First row: small-pen scenarios (S); second row: large-pen scenarios (L). First column: individual treatment only (I); second column: with collective treatment enabled during fattening (C).

As clinical illness is also responsible for a reduction in weight gain, we also considered the trade-off between the cumulative duration of presence of animals with severe clinical signs during the whole 100-days period (using this as a proxy for disease impact) and the total number of antimicrobial doses used for treating detected BRD cases (Figure 7). Scenarios with large pens and low risk (L-LR-I, L-LR-C) clearly outperformed all other scenarios both regarding antimicrobial usage and BRD duration, followed by the low-risk scenarios in small pens (S-LR-I, S-LR-C). The cost of antibioprevention (HRA) in terms of antimicrobial usage was also very visible, but came with a positive effect on BRD impact compared to the same risk level without antibioprevention (HR), especially when only individual treatment was allowed.

**Figure 8:**
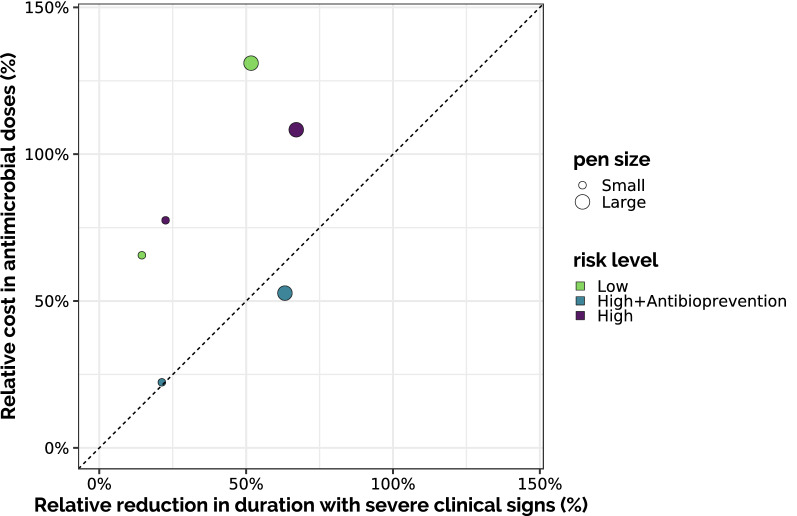
**Impact of collective treatment.** Each point represents the relative average additional consumption of antibiotics doses and relative average reduction in the cumulate disease duration when allowing collective treatment during fattening, compared to the same scenario with individual treatment only. The bisector (dashed line) represents theoretical situations when gaining X% of disease duration would require an additional X% of antimicrobial doses: for points above this line, the relative cost in antimicrobials induced by the collective treatment was higher than the relative gain in disease duration.

To assess more specifically the added value of collective treatments during fattening, we examined the trade-off between the relative reduction in the average cumulative duration with severe clinical signs when allowing a collective treatment during fattening and the relative increase of the average number of antimicrobial doses required to do so, compared to the same situation with individual treatments only (Figure 8). The dashed line represents situations where the relative “gain” (reducing disease duration) is the same as the relative antimicrobial “cost” (increasing doses): above the line, the relative cost is higher than the relative gain. In large pens, both relative costs and gains were higher than their counterparts in small pens. Also, the added value of collective treatments was higher for large high-risk pens than for low-risk ones (higher gain, lower cost). Yet, with this representation HRA scenarios appeared with a low antimicrobial cost, but this was quite misleading, as the cost for collective treatment during fattening was diluted by the cost of antibioprevention.

**Figure 9:**
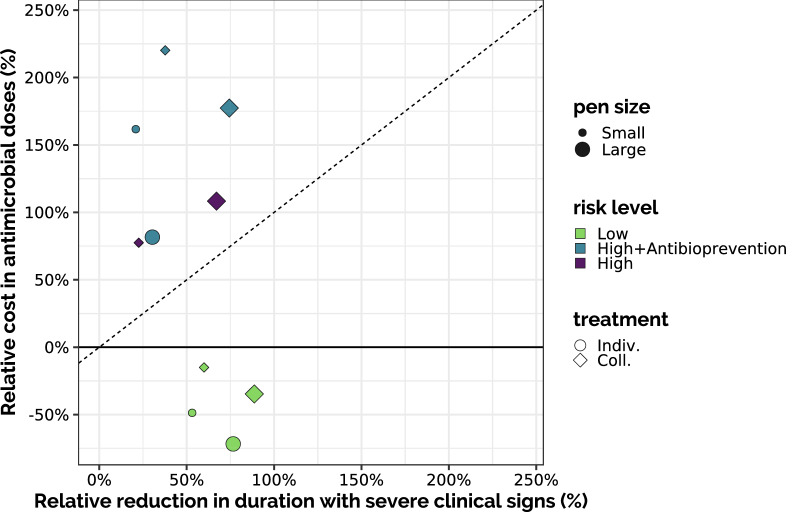
**Comparisons with the high-risk scenario with individual treatment only.** Each point represents, for a given pen size, the relative average additional consumption of antibiotics doses and relative average reduction in the cumulate disease duration for each scenario, compared to the scenario with high risk level and individual treatment only (for the same pen size). The bisector (dashed line) represents theoretical situations when gaining X% of disease duration would require an additional X% of antimicrobial doses.

Similarly, Figure 9 presents, for both pen sizes, a comparison between individual treatment in high risk pens and all other scenarios. In large pens, collective treatment during fattening provided a higher gain than antibioprevention only, with a slightly higher cost. In small pens, the scenarios with antibioprevention appeared highly antimicrobial-consuming without a clear gain compared to the used of a collective treatment without prior antibioprevention. But, above all, this figure highlighted the interest of reducing the risk level at pen formation, as low-risk scenarios always outperformed other practices.

### Sensitivity analysis

**Figure 10:**
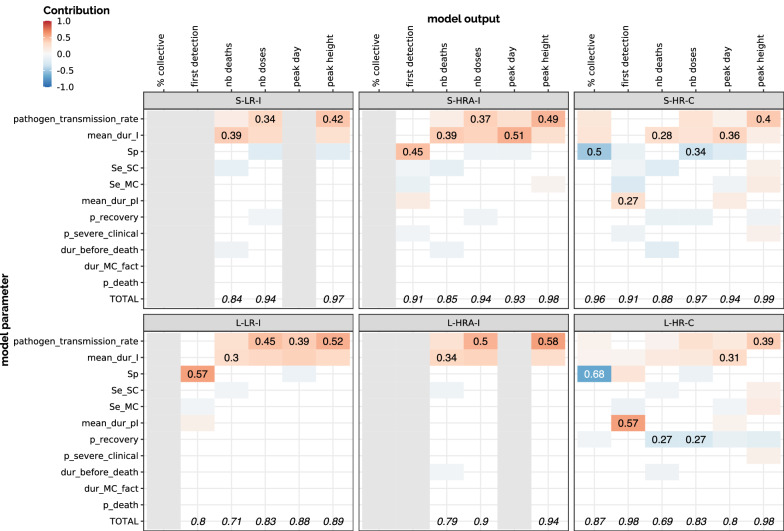
**Sensitivity analysis for 6 scenarios.** In both small (S) and large (L) pens, we considered the following scenarios: low risk (LR) or high risk with antibioprevention (HRA) with individual treatment only (I), vs high risk (HR) with collective treatment (C). For each scenario, we display the contribution (total sensitivity index calculated by an ANOVA, see Equation 4) of each parameter (one per line) to the variation of target outputs (one per column) when this contribution was over 5%. This contribution was made positive or negative depending on the sign of the correlation between parameter and output variations. For each output (i.e. for each column), the numeric values represent the effect of the most impactful parameter (on the corresponding line) and the sum of all contributions (part of the variations that is explained by the parameters chosen in the sensitivity analysis). Grey columns correspond to outputs that either were not relevant in the corresponding scenario (proportion of collective treatments in the individual-treatment scenarios) or could not be analysed because their distribution was not normal.

Results of the sensitivity analysis for LR-I, HRA-I and HR-C scenarios are summarized on Figure 10. It appeared that in all scenarios, both the pathogen transmission rate and the average duration of the infectious state (mean_dur_I) play a key role (with comparable weights) in the variability of outputs related to BRD dynamics and impact in terms of antimicrobial usage and mortality.

However, the specificity of detection appeared to play a major role in the date of first detection, except for high-risk scenarios where the first detection is mainly driven by the delay before asymptomatic carriers become infectious (mean_dur_pI). When collective treatments on high-risk pens are allowed, the specificity also impacts the proportion of pens where a collective treatment was actually triggered, which in turn affects the number of antimicrobial doses. Also, in the latter scenarios, the efficacy of antimicrobials (p_recovery) impacts the number of doses and the mortality, all the more in large pens. Interactions between parameters had little influence on the outputs and were thus not represented.

## Discussion

This model made it possible to explore BRD dynamics, as well as the morbidity, lethality and antimicrobial usage, in various combinations of farming practices, risk levels, and individual vs. collective treatment strategies, which are consistent with existing observations [12, 26, 28]. This required to incorporate two mechanisms involved in BRD, direct transmission and asymptomatic carriage of pathogens, and a fine-grained representation of how clinical signs appear and are detected. The main difference in farming practices lies in the contrasted size of pens. A larger size of livestock groups is often responsible for higher prevalence of respiratory diseases in several species [29–31], hence it could be expected that fattening the same number of beef calves was more risky in a single large pen than in several smaller pens. This was found indeed in the large-pen high-risk scenarios compared to the small-pen ones. Yet, our assumption that between-pen transmission could be neglected should be investigated further to clarify the interest of small pens as a risk mitigation strategy.

Also, it appeared that the use of antibioprevention strongly reduced morbidity and mortality, which has a positive impact on animal welfare as well as fattening performance. It indeed requires more overall antimicrobial doses, but the compromise in favour of reducing BRD impact is relevant and valuable for high-risk calves. In practice indeed, almost all high-risk pens are mitigated by antibioprenvention, which for instance represents about 25% of US cattle [22]. This also suggests to investigate the benefits of identifying relevant thresholds for collective treatments, i.e. the number of detected cases above which a collective treatment would be relevant to reduce disease impact and antimicrobial cost at the same time [32].

By contrast, the large-pen low-risk scenarios proved much more efficient than all the others, including the small-pen ones, in terms both of cumulative disease duration and of the number of antimicrobial doses. This prompts the consideration of a more thorough assessment of the level of risk associated with small pens, and more generally to enforce risk reduction at pen formation. Multiple factors are already known for being involved in BRD dynamics [17, 33–35] such as the diversity of origins and distance travelled [13, 36], making it possible to predict the risk level associated with each pen, even in small-pen systems [21]. In addition, methods have also been recently proposed to reduce the diversity of origins and travel distance of animals gathered in a same pen by reallocating trade movements in sorting facilities, in order to minimize the impact of BRD [37, 38]. They essentially consist in algorithms aiming at modifying the way calves are assigned to their destination batches, to reduce known risk factors. The combination of best practices in pen formation and systematic risk assessment could provide better BRD control strategies, enabling to focus on efficient detection methods for pens identified as high-risk.

The specificity of detection by mild or severe clinical signs, though assumed rather high in the model (0.9), appeared to have a substantial impact on the decision to implement collective treatments, when those were allowed. Indeed, they might have been triggered due to false positive animals, and nevertheless proved effective by wiping out infectious animals not yet detected. This issue is all the more crucial as the uncertainties on sensitivity and specificity estimates remain very large [15, 16], and this highlights the need to fill such knowledge gaps. It also suggests investigating alternative detection strategies, for instance based on individual sensors (e.g., ear tags, accelerometer collars, intraruminal thermoboluses…) or on collective monitoring (e.g., cough detection), to implement early or individualized strategies before pathogen spread.

Other reported BRD observations could be explained by effects that were not accounted for in this study, such as a variability in breeds, zootechnical conditions, or a difference in pathogens. Indeed, beef breeds used in fattening feedlots are not the same in all countries and could have a specific susceptibility to respiratory pathogens [39, 40], but also different capability to concealing clinical signs [25], which could impact disease dynamics and detection. Besides, small pens are generally fattened indoor, which facilitates pathogen spread, rather than in the open air. Finally, our assumption that the same “average” pathogen could be used for all scenarios is indeed questionable. BRD is intrinsically a multi-pathogen disease, and the exact prevalence of each pathogen, their possible interactions, as well as the diversity of strains, can be expected to change the dynamics of infection and disease severity [3, 41]. In this study we assumed an average pathogen to keep the model as simple as possible. However, in further study, model parameters reflecting microbiological characteristics (e.g., the mean duration of infectiousness and the pathogen transmission rate) could be made pathogen-specific to allow for comparisons between various pathogens.

## Supplementary Information

**Additional file 1.** Additional information on the processes involved in the model (section 1) and the whole content of the model (section 2).

**Additional file 2. BRD model in EMULSION format.** BRD model expressed within the modelling language used by the EMULSION simulation engine (based on YAML format).

## Data Availability

The datasets used to calibrate the model have been published in earlier studies [10, 12, 26]. EMULSION is an open source software which can be installed as a Python module (https://sourcesup.renater.fr/www/emulsion-public). The BRD model file (brd.yaml) is provided as Additional file 2.
