# Supplementary material for: Modelling the effects of antimicrobial metaphylaxis and pen size on bovine respiratory disease in high and low risk fattening cattle
Source: Vet Res. 2022 Oct 4;53:77. doi: 10.1186/s13567-022-01094-1 (PMC9531528; doi:10.1186/s13567-022-01094-1)
Supplement: Supplementary file 1 — Additional file 1. Additional information on the processes involved in the model (section 1) and the whole content of the model (section 2). [file 13567_2022_1094_MOESM1_ESM.doc]

# Additional file 1

Table of contents

[1 Processes of the model](#__RefHeading___Toc107864947)

[2 Complete EMULSION BRD model](#__RefHeading___Toc107864954)

# Processes of the model

In the model, five processes drive the states of individuals: infection (Additional file 1A), clinical signs (Additional file 1C), detection (Additional file 1D) and treatment (Additional file 1E). They are represented by a formalism broadly used in computer science, finite state machines, which is close to flow diagrams used by epidemiologists, with a higher expressiveness. Their textual description in the YAML format in the model file (section 2 below, or Additional file 2) is easy to read and can be automatically converted in a graphical representation by EMULSION.

To help understand the state machine diagrams, we present how the diagram summarising the infection process (Additional file 1A) relates to the state machine specification (Additional file 1B), extracted from the whole model (Additional file 2). More details on how state machines differ from flow diagrams are available in the documentation: <https://sourcesup.renater.fr/www/emulsion-public/pages/Modelling_principles.html>


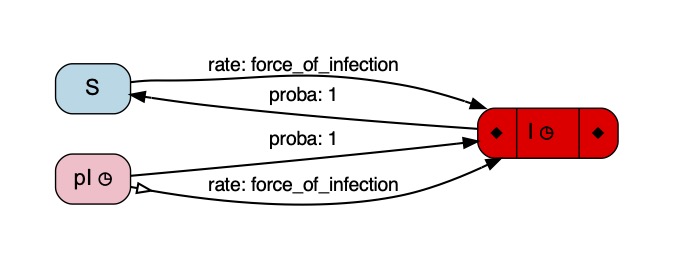


**Additional file 1A. State machine representing the infectious process at the individual scale.** Health states: susceptible (S), infectious (I), asymptomatic carrier (pI). After a delay (denoted by the clock), pI individuals eventually become infectious (hence, they have a probability of 1 to move to I after the delay) but can also become infectious at any moment (transition at the bottom with an open arrow at the beginning of the edge), due to contacts with infectious animals. Susceptible animals can become I only through contacts with other I. Actions are performed when entering or leaving I state (denoted by the lozenges), especially for controlling hyperthermia. After a delay, infectious individuals become susceptible again.

state_machines:

health_state:

desc: 'Description of the infectious process'

states:

**- S:**

**name: 'Susceptible'**

**desc: 'Suceptible to infection'**

**fillcolor: 'lightblue'**

- I:

name: 'Infectious'

desc: 'Infected and able to transmit pathogens to susceptible individuals [...]'

fillcolor: 'red'

duration: 'dur_I'

on_enter:

- set_var: infected

value: 1

[...]

on_exit:

- set_upper_var: pen_infection_duration

value: 'pen_infection_duration + duration_in_health_state'

[...]

**- pI:**

**name: 'pre-infectious'**

**desc: 'State for initializing animals that will eventually become infectious [...]'**

**fillcolor: 'pink'**

**duration: 'dur_pI'**

transitions:

- from: pI

to: I

proba: 1

desc: 'pre-infectious animals become infectious after a delay'

- from: pI

to: I

escape: yes

rate: 'force_of_infection'

desc: 'before the end of the carriage period, pre-infectious animals become infectious due to contacts with other infectious animals'

- from: S

to: I

rate: 'force_of_infection'

desc: 'classical infection of susceptible individuals due to conctacts with other infectious animals'

- from: I

to: S

proba: 1

desc: 'infectious animals naturally return to susceptible state after duration dur_I'

Individual duration in pI state (sampled in dur_pI distribution).

All individuals that have spent their duration in pI move to I (proba: 1). In the meanwhile (escape: yes), they can also become I due to infection, at a rate determined by the force of infection (specified in ‘parameters’)

Individual duration in I state (sampled in dur_I distribution , defined in the ‘parameters’ section in whole model).

Actions performed by individuals when they enter or leave I state

This transition has proba: 1 but only individuals that have spent their duration in I can cross

**Additional file 1B. Annotated description of the “health_state” state machine.** This part of the BRD model produces the state machine diagram of Additional file 1A.


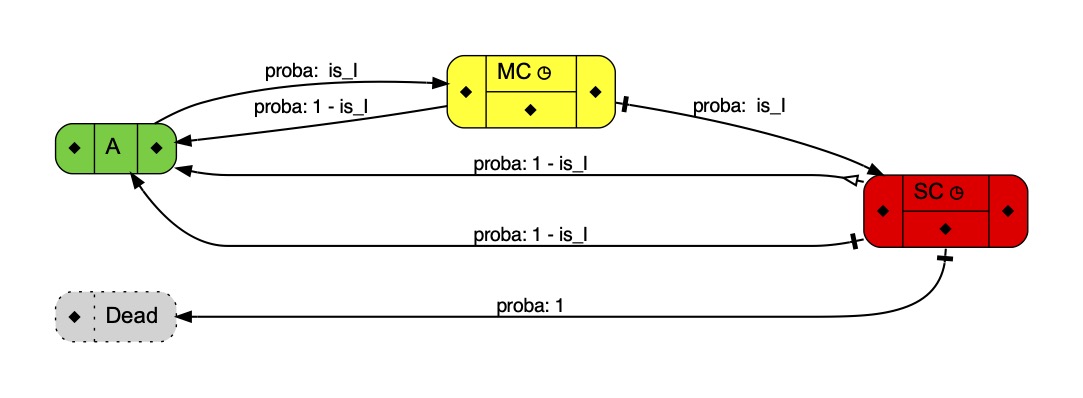


**Additional file 1C. State machine representing the onset of clinical signs.** States: asymptomatic (A), mildly symptomatic (MildC) with low detection sensitivity, symptomatic with severe and more sensitive clinical signs (SC), dead (Dead). Mild clinical signs appear with infection, then can either evolve towards severe clinical signs, or disappear when infection is over. When entering SC state, individuals are subject to a random trial to determine whether they will eventually die from BRD or return to A (denoted by the vertical bar on the transitions from SC).


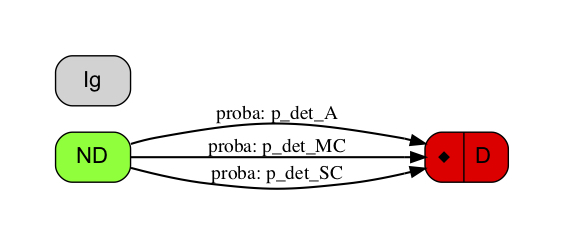


**Additional file 1D. State machine representing BRD detection.** States: undetected (ND), detected (D), Ignored (Ig). Detection probabilities depend on the clinical status, based on the specificity for A (false positive), and on the sensitivity for mild and severe clinical signs for MC and SC, respectively. State “Ig” is set when a treatment fails after the maximum allowed number of doses: in that case, the animal is no longer considered for receiving further treatments.


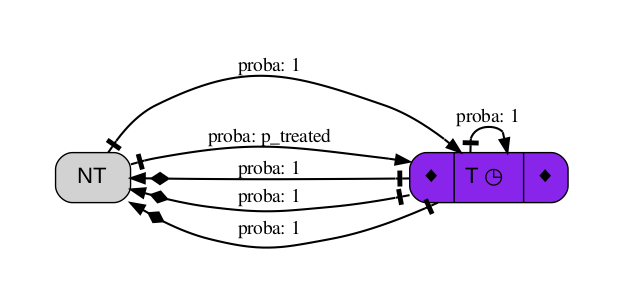


**Additional file 1E. State machine representing the treatment protocol.** States: not under treatment (NT), under treatment (T). Detected animals become T with a probability p_treated and receive a first antimicrobial dose. A random trial determines the outcome of the treatment, which will be effective after a constant delay (48 h). Animals with a successful treatment or returned to asymptomatic in the meanwhile return to NT. Otherwise, they are re-treated until receiving the maximum number of doses (here, 3), then the treatment is abandoned, and the animal will not be re-treated anymore. For each of these disjoint conditions, the decision is deterministic (hence ‘proba: 1’).

# Complete EMULSION BRD model

The following file (Additional file 2, to be renamed as “brd.yaml”) is meant to be run with the EMULSION framework (version 1.2b11 or later) which can be installed according to the instructions provided on the software webpage: <https://sourcesup.renater.fr/www/emulsion-public/>. The syntax of the EMULSION modelling language is also fully described there.

Assuming EMULSION installed properly, running a scenario (defined by specific parameter values), e.g. L-HR-C for 100 stochastic repetitions, requires the following command:

emulsion run brd.yaml -r 100 --output-dir L-HR-C -p pen_size=100 -p initial_prevalence=0.2 -p use_antibioprevention=0 -p use_metaphylaxis=1 -p metaphylaxis_threshold=0.15

Simulation results are stored in directory ‘L-HR-C’ in a CSV file named ‘counts.csv’.

---

# _____ _

# / ____| | |

# | | __ ___ _ __ ___ _ __ __ _| |

# | | |_ |/ _ \ '_ \ / _ \ '__/ _` | |

# | |__| | __/ | | | __/ | | (_| | |

# \_____|\___|_| |_|\___|_| \__,_|_|

# _____ __ _ _

# |_ _| / _| | | (_)

# | | _ __ | |_ ___ _ __ _ __ ___ __ _| |_ _ ___ _ __

# | | | '_ \| _/ _ \| '__| '_ ` _ \ / _` | __| |/ _ \| '_ \

# _| |_| | | | || (_) | | | | | | | | (_| | |_| | (_) | | | |

# |_____|_| |_|_| \___/|_| |_| |_| |_|\__,_|\__|_|\___/|_| |_|

# NAME OF THE DISEASE

model_name: BRD_FR_US_VetRes

## information

model_info:

authors:

- 'Sébastien Picault, INRAE, Oniris, BIOEPAR, 44300 Nantes, France'

- 'Pauline Ezanno, INRAE, Oniris, BIOEPAR, 44300 Nantes, France'

- 'Sébastien Assié, INRAE, Oniris, BIOEPAR, 44300 Nantes, France'

contributors:

- 'Brad White, Beef Cattle Institute, Kansas State University, USA'

- 'David Amrine, Beef Cattle Institute, Kansas State University, USA'

publications:

- 'Picault S, Ezanno P, Smith K, White B, Amrine D, Assié S. 2022 (under review). Modelling the effects of antimicrobial metaphylaxis and pen size on Bovine Respiratory Disease in high and low risk fattening cattle. Submitted to Veterinary Research (ModAH series)'

# _______ _

# |__ __(_)

# | | _ _ __ ___ ___

# | | | | '_ ` _ \ / _ \

# | | | | | | | | | __/

# |_| |_|_| |_| |_|\___|

# TIME INFORMATION

time_info:

time_unit: hours

delta_t: 12

total_duration: '100*24'

# _ _

# | | | |

# | | _____ _____| |___

# | | / _ \ \ / / _ \ / __|

# | |___| __/\ V / __/ \__ \

# |______\___| \_/ \___|_|___/

# LEVELS OF AGENTS

levels:

beef_calves:

desc: 'level of individuals (young calves, if not males only, assuming no difference between males and females in the infection processes)'

default_prototype: default_beef_calf

pen:

desc: 'level of the pen'

contains:

- beef_calves

aggregation_type: hybrid

default_prototype: default_pen

aggregate_vars:

- name: pen_nb_alive_treated_distinct

collect: 'treated'

operator: 'sum'

- name: pen_nb_alive_infected_distinct

collect: 'infected'

operator: 'sum'

- name: pen_nb_alive_detected_distinct

collect: 'detected'

operator: 'sum'

- name: pen_nb_doses_alive

collect: 'nb_doses_total'

operator: 'sum'

- name: pen_nb_treated_only_once

collect: 'treated_only_once'

operator: 'sum'

# _____

# | __ \

# | |__) | __ ___ ___ ___ ___ ___ ___ ___

# | ___/ '__/ _ \ / __/ _ \/ __/ __|/ _ \/ __|

# | | | | | (_) | (_| __/\__ \__ \ __/\__ \

# |_| |_| \___/ \___\___||___/___/\___||___/

# LIST OF PROCESSES (IN ORDER)

processes:

beef_calves:

- health_state: infection

- clinical_state: clinical_signs

- detection_state: detection

- treat_state: treatment

# _____

# / ____|

# | | __ _ __ ___ _ _ _ __ ___

# | | |_ | '__/ _ \| | | | '_ \/ __|

# | |__| | | | (_) | |_| | |_) \__ \

# \_____|_| \___/ \__,_| .__/|___/

# | |

# |_|

# DESCRIPTION OF HOW TO GROUP INDIVIDUALS

grouping:

pen:

infection: [health_state]

treatment: [treat_state]

clinical_signs: [clinical_state, health_state]

detection: [detection_state, clinical_state]

# _____ _ _ __ __ _ _

# / ____| | | | | \/ | | | (_)

# | (___ | |_ __ _| |_ ___ | \ / | __ _ ___| |__ _ _ __ ___ ___

# \___ \| __/ _` | __/ _ \ | |\/| |/ _` |/ __| '_ \| | '_ \ / _ \/ __|

# ____) | || (_| | || __/ | | | | (_| | (__| | | | | | | | __/\__ \

# |_____/ \__\__,_|\__\___| |_| |_|\__,_|\___|_| |_|_|_| |_|\___||___/

# DESCRIPTION OF THE STATE MACHINES INVOLVED IN THE MODEL

state_machines:

#--------------------------------

# INFECTION

#--------------------------------

health_state:

desc: 'Description of the infectious process'

states:

- S:

name: 'Susceptible'

desc: 'Suceptible to infection'

fillcolor: 'lightblue'

- I:

name: 'Infectious'

desc: 'Infected and able to transmit pathogens to susceptible individuals. The duration of infection (in the absence of control) is sampled in distribution dur_I.'

fillcolor: 'red'

duration: 'dur_I'

on_enter:

- set_var: infected

value: 1

on_exit:

- set_upper_var: pen_infection_duration

value: 'pen_infection_duration + duration_in_health_state'

- set_var: nb_consecutive_doses

value: 0

- pI:

name: 'pre-infectious'

desc: 'State for initializing animals that will eventually become infectious. This represent an asymptomatic carriage of pathogens, which can lead to the onset of infectiousness just after entering the pen or later. Pre-infectious individuals can however be infected (and become infectious) due to contacts with infectious individuals'

fillcolor: 'pink'

duration: 'dur_pI'

transitions:

- from: pI

to: I

proba: 1

desc: 'pre-infectious animals become infectious after a delay'

- from: pI

to: I

escape: yes

rate: 'force_of_infection'

desc: 'before the end of the carriage period, pre-infectious animals become infectious due to contacts with other infectious animals'

- from: S

to: I

rate: 'force_of_infection'

desc: 'classical infection of susceptible individuals due to conctacts with other infectious animals'

- from: I

to: S

proba: 1

desc: 'infectious animals naturally return to susceptible state after duration dur_I'

#--------------------------------

# CLINICAL SIGNS

#--------------------------------

clinical_state:

desc: 'Succession of clinical states experienced by infected animals.'

states:

- A:

name: 'Asymptomatic'

desc: 'Animal with no clinical signs'

fillcolor: 'limegreen'

on_enter:

- set_var: evolve_to_SC

value: 0

# the evolution from mild to severe clinical signs will

# be determined each time the animal enters MC

on_exit:

- set_upper_var: pen_nb_true_negative

value: 'pen_nb_true_negative + is_ND'

# if the detected animal leaves the asymptomatic state

# without detection, increase the number of true negative

- MC:

name: 'Mild clinical signs'

desc: 'Animal infected, which may have mild clinical signs which are difficult to detect. Some of these animals will eventually develop severe clinical signs with probability p_severe_clinical (assumed independent from infection duration), after a given duration'

fillcolor: 'yellow'

duration: 'dur_MC'

on_enter:

- set_var: evolve_to_SC

value: 'OR(random_bool(p_severe_clinical))'

# determine whether or not an animal with mild clinical

# signs will eventually develop severe clinical signs

on_stay:

- set_upper_var: pen_nb_MC_missed

value: 'pen_nb_MC_missed + is_ND * Eq(time,total_duration)'

# if no detection occurred before the end of the simulation, count as false negative for MC

on_exit:

- set_upper_var: pen_nb_MC_missed

value: 'pen_nb_MC_missed + is_ND'

# if no detection occurred when leaving MC (either to SC or A), count as false negative for MC

- set_upper_var: pen_mild_clinical_duration

value: 'pen_mild_clinical_duration + duration_in_clinical_state'

# record total duration of MC state

- SC:

name: 'Severe clinical signs'

desc: 'Animals with severe clinical signs (depression, anorexia) highly susceptible to trigger detection. A small proportion of animals with severe clinical signs will eventually die from BRD after dur_before_death'

fillcolor: 'red'

# assign default duration which is the time before dying from severe clinical signs

duration: dur_before_death

on_enter:

- set_var: evolve_to_death

value: 'OR(evolve_to_death, random_bool(p_death))'

# "OR" operator to ensure that animals tagged previously

# as going to die cannot escape their fate

- set_var: _time_to_exit_clinical_state

value: 'IfThenElse(evolve_to_death, _time_to_exit_clinical_state, 0)'

# if severe clinical signs are not expected to lead to

# death, reset duration to 0 to allow animals to go back

# to A as soon as they go back to S

on_stay:

- set_upper_var: pen_nb_SC_missed

value: 'pen_nb_SC_missed + is_ND * Eq(time,total_duration)'

# if no detection occurred before the end of the simulation, count as false negative for sC

on_exit:

- set_upper_var: pen_nb_SC_missed

value: 'pen_nb_SC_missed + is_ND'

# if no detection occurred when leaving SC (either to A or Dead), count as false negative for SC

- set_upper_var: pen_severe_clinical_duration

value: 'pen_severe_clinical_duration + duration_in_clinical_state'

# record total duration of SC state

- Dead:

name: 'Dead'

desc: 'Animals that die from BRD. When animals die, record durations if still I or H or T'

fillcolor: 'lightgray'

autoremove: yes

on_enter:

- record_change: pen_nb_deaths

- set_var: treated_only_once

value: 0

- set_upper_var: pen_infection_duration

value: 'pen_infection_duration + duration_in_health_state * is_I'

- set_upper_var: pen_nb_dead_treated_distinct

value: 'pen_nb_dead_treated_distinct + treated'

- set_upper_var: pen_nb_dead_infected_distinct

value: 'pen_nb_dead_infected_distinct + infected'

- set_upper_var: pen_nb_dead_detected_distinct

value: 'pen_nb_dead_detected_distinct + detected'

- set_upper_var: pen_nb_doses_dead

value: 'pen_nb_doses_dead + nb_doses_total'

transitions:

- from: A

to: MC

proba: ' is_I'

desc: 'Infected animals develop mild (undetectable) clinical signs, some of them will eventually develop severe clinical signs later'

- from: MC

to: SC

cond: evolve_to_SC

proba: ' is_I'

desc: 'Animals develop detectable (severe) clinical signs after dur_MC if they were marked to develop severe clinical signs (when entering C) and still infected'

- from: MC

to: A

proba: '1 - is_I'

desc: 'Animals return to Asymptomatic after dur_MC if they are not infected anymore'

- from: SC

to: Dead

cond: ' evolve_to_death'

proba: 1

desc: 'the few animals that have very severe clinical signs will eventually die from BRD (if not successfully treated in the meanwhile)'

- from: SC

to: A

escape: 'NOT(evolve_to_death)'

proba: '1 - is_I'

desc: 'animals that are not marked as going to die return to Asymptomatic as soon as infection is over even before duration in SC'

- from: SC

to: A

cond: 'NOT(evolve_to_death)'

proba: '1 - is_I'

desc: 'animals that are not marked as going to die return to Asymptomatic as soon as infection is over'

#--------------------------------

# DETECTION

#--------------------------------

detection_state:

desc: 'Description of the state of individuals regarding detection'

states:

- ND:

name: 'Undetected'

desc: 'Animal considered healthy by the farmer (whatever its real health state).'

fillcolor: 'green'

- D:

name: 'Detected'

desc: 'Animal considered infected by the farmer (whatever its real health state).'

fillcolor: 'red'

on_enter:

- set_var: detected

value: 1

- set_upper_var: pen_nb_false_positive

value: 'pen_nb_false_positive + is_A'

# if the detected animal was actually asymptomatic ->

# increase the number of false positive

- set_upper_var: pen_nb_MC_detections

value: 'pen_nb_MC_detections + is_MC'

# if the detected animal has mild clinical signs

# increase the number of this detection method

- set_upper_var: pen_nb_SC_detections

value: 'pen_nb_SC_detections + is_SC'

# if the detected animal has severe clinical signs

# increase the number of this detection method

- set_upper_var: pen_prev_cumulate_incidence

value: 'pen_cumulate_incidence'

- record_change: pen_cumulate_incidence

- set_upper_var: pen_previous_half_day_incidence

value: 'pen_half_day_incidence'

- set_upper_var: pen_half_day_incidence

value: 'pen_cumulate_incidence - pen_prev_cumulate_incidence'

- set_upper_var: pen_time_first_detection

value: 'IfThenElse(pen_time_first_detection < 0, time, pen_time_first_detection)'

- set_upper_var: pen_do_collective_treatment

value: 'MAX(pen_do_collective_treatment, compute_collective_treatment_decision)'

- set_upper_var: pen_previous_half_day_incidence

value: 'IfThenElse(pen_do_collective_treatment * use_daily_incidence, 0, pen_previous_half_day_incidence)'

- set_upper_var: pen_half_day_incidence

value: 'IfThenElse(pen_do_collective_treatment * use_daily_incidence, 0, pen_half_day_incidence)'

- set_upper_var: pen_cumulate_incidence

value: 'IfThenElse(pen_do_collective_treatment * (1 - use_daily_incidence), 0, pen_cumulate_incidence)'

# if collective treatment decided, ensure that next incidence will be 0

# (to avoid restarting collective treatment over and over) otherwise do not change anything

- Ig:

name: 'Ignored'

desc: 'Animal previously detected but which successive treatments failed, hence no more considered for further treatments'

transitions:

- from: ND

to: D

proba: p_det_A

desc: 'False positive detection due to the lack of specificity'

- from: ND

to: D

proba: p_det_MC

desc: 'Detection of an animal with mild clinical signs'

- from: ND

to: D

proba: p_det_SC

desc: 'Detection of an animal with severe clinical signs'

#--------------------------------

# TREATMENTS

#--------------------------------

treat_state:

desc: 'Description of treatment protocol'

states:

- NT:

name: 'Non Treated'

desc: 'Non treated animal'

fillcolor: 'lightgray'

- T:

name: Treated

desc: 'Treated animal'

fillcolor: 'purple'

duration: dur_T

on_enter:

- set_var: treated

value: 1

- set_var: nb_consecutive_doses

value: '1 + nb_consecutive_doses'

- set_var: nb_doses_total

value: '1 + nb_doses_total'

- set_var: treated_only_once

value: 'Eq(nb_doses_total, use_antibioprevention + 1)'

- set_var: treatment_successful

value: 'random_bool(p_recovery)'

on_exit:

- set_upper_var: pen_do_collective_treatment

value: 0

transitions:

- from: NT

to: T

cond: 'AND(NOT(is_Ig), pen_do_collective_treatment)'

proba: 1

desc: 'When a collective treatment is implemented, treat all animals not currently treated unless they already received the maximum number of doses'

- from: NT

to: T

cond: 'AND(NOT(pen_do_collective_treatment), is_D, StrictLessThan(nb_consecutive_doses, nb_doses_max))'

proba: p_treated

desc: 'Treat individual animals as the result of individual detection. Avoid to re-treat animals which are still detectable but already got the max number of treatments (such animals stay in NT and will be re-detectable after they return to S at the natural end of disease) or animals that have been already treated even some time ago but will obviously die from BRD'

- from: T

to: NT

cond: 'is_A'

proba: 1

on_cross:

- become: healthy # to ensure that pI -> S

- set_var: nb_consecutive_doses

value: 0

- become: undetected # temporarily no more considered for treatments

desc: 'stop treatment after duration dur_T if the animal has no more clinical signs (recovery occurred before end of treatment)'

- from: T

to: NT

cond: 'AND(OR(is_SC, is_MC), treatment_successful)'

proba: 1

on_cross:

- become: healthy # I -> S (which induces H -> N)

- set_var: nb_consecutive_doses

value: 0

- become: undetected

desc: 'recovery occurs after treatment of duration dur_T assuming the treatment was successful'

- from: T

to: T

cond: 'AND(OR(is_SC, is_MC), NOT(treatment_successful), StrictLessThan(nb_consecutive_doses, nb_doses_max))'

proba: 1

desc: 'restart treatment after duration dur_T assuming no recovery occurred despite treatment, and number of doses below the authorized threshold'

- from: T

to: NT

cond: 'AND(OR(is_SC, is_MC), NOT(treatment_successful), Eq(nb_consecutive_doses, nb_doses_max))'

proba: 1

on_cross:

- become: ignored

desc: 'if no recovery occurred after the maximum number of treatments: abandon (do not reset the number of doses received to avoid restarting treatment immediately - this will be done when leaving I state)'

# _____ _

# | __ \ | |

# | |__) |_ _ _ __ __ _ _ __ ___ ___| |_ ___ _ __ ___

# | ___/ _` | '__/ _` | '_ ` _ \ / _ \ __/ _ \ '__/ __|

# | | | (_| | | | (_| | | | | | | __/ || __/ | \__ \

# |_| \__,_|_| \__,_|_| |_| |_|\___|\__\___|_| |___/

parameters:

# --------------------------------

# CLINICAL SIGNS PARAMETERS

# --------------------------------

dur_MC:

desc: 'Distribution of the durations of mild clinical signs caused by infection, before the apparition of severe clinical signs likely to trigger treatment (hours)'

value: 'dur_MC_fact * random_beta(1.08, 1.69)*111 + 12'

source: 'Timsit et al 2011, fig. 4. Min-Max = 12-123; Approx. quartiles: 42, 51, 75. Estimated beta distribution from Q2 and Q3: alpha=1.08, beta=1.69'

mean_dur_MC:

desc: 'average value of the dur_MC distribution (hours) - beware: to update according to dur_MC'

value: 55

dur_MC_expected:

desc: 'expected value of dur_MC, which is mean_dur_MC for individuals that develop severe clinical signs, and mean_dur_I for the others'

value: 'p_severe_clinical * dur_MC_fact * mean_dur_MC + (1 - p_severe_clinical) * mean_dur_I'

mean_dur_SC:

desc: 'average value of the duration of severe clinical signs for animals that will not die from BRD (hours)'

value: 'mean_dur_I - mean_dur_MC'

dur_SC_expected:

desc: 'expected value of dur_MC, which is mean_dur_SC for individuals that will not die from BRD, and dur_before_death for the others'

value: '(1 - p_death) * mean_dur_SC + p_death * dur_before_death'

dur_A_expected:

desc: 'expected value of the duration of asymptomatic state, which is longer that the simulation time (range of values: about 150 days on average in [White and Rentner 2009], but 10 months in [Assié et al 2009])'

value: '200 * 24'

p_severe_clinical:

desc: 'probability that an animal with mild clinical signs eventually develops severe clinical signs'

value: 0.5

source: 'expert opinion'

p_death:

desc: 'probability that an animal with severe clinical signs eventually dies from BRD'

value: 0.05

source: 'expert opinion'

dur_before_death:

desc: 'distribution of the durations of severe clinical signs before death (only for animals that will die from BRD)'

value: '10*24'

source: 'expert opinion'

# --------------------------------

# EPIDEMIOLOGICAL PARAMETERS

# --------------------------------

dur_I:

desc: 'distribution of durations in the infectious state'

# value: 'poisson(mean_dur_I)'

value: 'random_gamma(dur_I_shape, mean_dur_I/dur_I_shape)'

source: 'trying gamma distribution which is equivalent to N(=10) consecutive geometric distribution which slowly converges towards a constant distribution - quartiles 92.7, 116, 143 (h)'

dur_I_shape:

desc: 'shape of the gamma distribution for I durations - high values: close to constant, low values: long tail'

value: 10

mean_dur_I:

desc: 'average duration in infectious states (in hours)'

value: '5*24'

source: 'expert opinion for an average pathogen'

dur_pI:

desc: 'Distribution of the durations of asymptomatic carriage of BRD pathogens, shifted by the use of long-acting antimicrobials when antibioprevention is performed'

value: 'default_dur_pI + use_antibioprevention * shifted_dur_pI'

mean_dur_pI:

desc: 'average duration in carriage state (in hours)'

value: '3*24'

source: 'previously calibrated for scenarios: FR/FRref: 2*24 ; USLR/USHR/USHRM: 6*24 — set to arbitrary value 3*24 in generic study'

dur_pI_shape:

desc: 'shape of the gamma distribution for pI durations - high values: close to constant, low values: long tail'

value: 5

default_dur_pI:

desc: 'Default distribution of the durations of asymptomatic carriage of BRD pathogens'

value: 'random_gamma(dur_pI_shape, mean_dur_pI/dur_pI_shape)'

source: 'testing alternative distributions : gamma law to have a few high values'

shifted_dur_pI:

desc: 'Shift in the distribution of the durations of asymptomatic carriage of BRD pathogens due to the use of long-acting antimicrobials'

value: 'random_gamma(shift_dur_pI_shape, mean_shift_dur_pI/shift_dur_pI_shape)'

mean_shift_dur_pI:

desc: 'average duration of the delay induced by long-acting antimicrobials'

value: '24 * 15'

source: 'calibration'

shift_dur_pI_shape:

desc: 'shape of the gamma distribution for pI shift'

value: 20

pathogen_transmission_rate:

desc: 'average value of individual transmission rate (per hour)'

value: '0.008'

source: 'calibrated for FR/US system'

force_of_infection:

desc: 'The force of infection experienced by each susceptible animal depends on the sum of individual infectiousness and exposure to general microbism of the farm'

value: 'pathogen_transmission_rate * total_I / total_pen + external_risk'

source: 'assuming frequency-dependent force of infection'

external_risk:

desc: 'contribution of the microbism of the herd (outside of the pen) to the force of infection. Set to 0 to represent isolated pens'

value: 0

pen_nb_infected_distinct:

desc: 'total number of distinct animals infected at least once in the pen over time'

value: 'pen_nb_alive_infected_distinct + pen_nb_dead_infected_distinct'

pen_nb_detected_distinct:

desc: 'total number of distinct animals detected at least once in the pen over time'

value: 'pen_nb_alive_detected_distinct + pen_nb_dead_detected_distinct'

#--------------------------------

# DETECTION

#--------------------------------

Se_MC:

desc: 'sensitivity of BRD detection based on mild clinical signs'

value: 0.3

source: 'Timsit et al 2016 - TODO discuss'

Se_SC:

desc: 'sensitivity of BRD detection based on severe clinical signs'

value: 0.6

source: 'White & Rentner 2009 - TODO discuss'

Sp:

desc: 'specificity of BRD detection assuming no difference between MC/SC'

value: 0.9

source: 'Timsit et al 2016 - TODO discuss'

p_det_MC:

desc: 'probability to detect a BRD case with mild clinical signs, each step (calculated from the sensitivity, the average duration in MC state, and the duration of one time step)'

value: 'is_MC * (1 - (1 - Se_MC)**(1 / dur_MC_expected))'

p_det_SC:

desc: 'probability to detect a BRD case with severe clinical signs, each step (calculated from the sensitivity, the average duration in SC state for animals not going to die from BRD, i.e. actually the average duration of infection minus the average duration with mild clinical signs)'

value: 'is_SC * (1 - (1 - Se_SC)**(1 / dur_SC_expected))'

p_det_A:

desc: 'probability to detect a false positive BRD case though asymptomatic, each step (calculated from the specificity and the duration over which this specificity was calculated'

value: 'is_A * (1 - Sp ** (1 / dur_A_expected))'

pen_daily_incidence:

desc: 'daily incidence at pen scale calculated from detections'

value: 'pen_half_day_incidence + pen_previous_half_day_incidence'

#--------------------------------

# TREATMENT

#--------------------------------

dur_T:

desc: 'Constant duration before assessing the efficacy of the treatment (hours)'

value: 48

source: 'average treatment protocol (discussed with professors in veterinary medicine)'

p_treated:

desc: 'probability that a deteced animal is treated'

value: 1

source: 'expert opinion'

p_recovery:

desc: 'probability that a treated animal recovers (for 1 dose)'

value: '0.8'

source: 'treatment protocol discussed with professors in veterinary medicine'

nb_doses_max:

desc: 'maximal number of consecutive doses for the same animal'

value: 3

source: 'treatment protocol discussed with professors in veterinary medicine'

use_antibioprevention:

desc: 'Boolean to indicate whether antibioprevention is used of not. With antibioprevention, all animals in the pen start with one dose (but initial_prevalence must be adjusted subsequently to account for treatment, e.g. * (1 - p_recovery))'

value: 0

pen_nb_treated_distinct:

desc: 'total number of distinct animals treated at least once in the pen over time'

value: 'pen_nb_alive_treated_distinct + pen_nb_dead_treated_distinct'

pen_nb_doses:

desc: 'total number of doses given at pen scale over time'

value: 'pen_nb_doses_alive + pen_nb_doses_dead'

use_metaphylaxis:

desc: 'Boolean to indicate whether metaphylaxis i.e. collective treatment during fattening is used. With metaphylaxis, when the incidence (daily or cumulate depending on pen size) reaches a given threshold, all animals get treated immediately'

value: 0

metaphylaxis_threshold:

desc: 'threshold to trigger collective treatment'

value: 0.15

use_daily_incidence:

desc: 'Boolean to indicate the method for deciding to implement a collective treatment. 1: implement when daily incidence > metaphylaxis_threshold ; 0: implement when cumulate incidence > metaphylaxis_threshold'

value: 0

compute_collective_treatment_decision:

desc: 'decide whether a collective treatment must be done or not - which can be triggered either by a daily incidence threshold, or by a cumulate incidence threshold'

value: 'use_metaphylaxis *

(IfThenElse(use_daily_incidence,

pen_daily_incidence,

pen_cumulate_incidence) >= metaphylaxis_threshold * total_pen)'

#--------------------------------

# INITIAL CONDITIONS

#--------------------------------

pen_size:

desc: 'initial size of the pen'

value: 100

source: 'French system: Assie, 10 animals - US system: White & Amrine, 100 animals'

initial_prevalence:

desc: 'initial proportion of (pre-)infected animals'

value: 0.1

source: 'calibrated values for scenarios: FR/FRref 0.1 ; USLR: 0.01; USHR: 0.14; USHRM: 0.06'

# --------------------------------

# SENSITIVITY ANALYSIS

# --------------------------------

dur_MC_fact:

desc: 'multiplicative factor used to modulate dur_MC (esp. in sensitivity analysis)'

value: 1

# _____ _ _

# / ____| | | |

# | (___ | |_ __ _| |_ _____ ____ _ _ __ ___

# \___ \| __/ _` | __/ _ \ \ / / _` | '__/ __|

# ____) | || (_| | || __/\ V / (_| | | \__ \

# |_____/ \__\__,_|\__\___| \_/ \__,_|_| |___/

# VARIABLES OF THE AGENTS

statevars:

#--------------------------------

# AGGREGATE VARIABLES AT PEN SCALE

#--------------------------------

pen_time_first_detection:

desc: 'date of first detection in the pen'

pen_infection_duration:

desc: 'cumulate infection duration at pen scale'

pen_mild_clinical_duration:

desc: 'cumulate duration of the presence of animals with mild clinical signs in the pen'

pen_severe_clinical_duration:

desc: 'cumulate duration of the presence of animals with severe clinical signs in the pen'

pen_nb_alive_treated_distinct:

desc: 'total number of distinct animals treated in the pen over time'

pen_nb_dead_treated_distinct:

desc: 'total number of distinct animals that were treated in the pen at least once but died'

pen_nb_alive_infected_distinct:

desc: 'total number of distinct animals infected at least once in the pen over time'

pen_nb_dead_infected_distinct:

desc: 'total number of distinct animals infected at least once in the pen over time but died'

pen_nb_alive_detected_distinct:

desc: 'total number of distinct animals detected at least once in the pen over time'

pen_nb_dead_detected_distinct:

desc: 'total number of distinct animals detected at least once in the pen over time but died'

pen_nb_doses_alive:

desc: 'total number of doses given at pen scale over time'

pen_nb_doses_dead:

desc: 'total number of doses given to animals that died'

pen_nb_deaths:

desc: 'total number of dead animals over time'

pen_nb_MC_detections:

desc: 'total number of detections based on mild clinical signs, over time'

pen_nb_MC_missed:

desc: 'total number of undetected animals though having mild clinical signs, over time'

pen_nb_SC_detections:

desc: 'total number of detections based on severe clinical signs, over time'

pen_nb_SC_missed:

desc: 'total number of undetected animals though having severe clinical signs, over time'

pen_nb_false_positive:

desc: 'total number of false positive detections (asymptomatic animals considered diseased), over time'

pen_nb_true_negative:

desc: 'total number of true negative animals (asymptomatic animals never detected while asymptomatic), over time'

pen_cumulate_incidence:

desc: 'total number of detected animals (true or false positive), over time'

pen_prev_cumulate_incidence:

desc: 'total number of detected animals (true or false positive) until previous time step, over time'

pen_half_day_incidence:

desc: 'total number of detected animals (true or false positive) during the current time step'

pen_previous_half_day_incidence:

desc: 'total number of detected animals (true or false positive) during the previous time step'

pen_do_collective_treatment:

desc: 'boolean to indicate whether a collective treatment is currently ongoing or not'

#--------------------------------

# INFECTION (Animal Level)

#--------------------------------

_time_to_exit_health_state:

desc: 'time step after which agents are allowed to leave current health state (EMULSION built-in)'

# infection_duration:

# desc: 'total duration of infection of an animal over time'

infected:

desc: 'boolean, 1 if animal infected at least once, 0 otherwise'

#--------------------------------

# CLINICAL SIGNS

#--------------------------------

evolve_to_death:

desc: 'indicate whether or not an animal with severe clinical signs will eventually die from BRD'

evolve_to_SC:

desc: 'indicate whether or not an animal with mild clinical signs will eventually develop severe clinical signs if still infected after dur_MC'

_time_to_exit_clinical_state:

desc: 'time step after which agents are allowed to leave current clinical state (EMULSION built-in)'

#--------------------------------

# DETECTION

#--------------------------------

detected:

desc: 'boolean, 1 if animal detected at least once, 0 otherwise'

#--------------------------------

# TREATMENT

#--------------------------------

treated:

desc: 'boolean value, 1 if animal treated at least once, 0 otherwise'

nb_consecutive_doses:

desc: 'number of consecutive doses given to the animal during the same episode'

nb_doses_total:

desc: 'total number of doses given to the considered animal'

treated_only_once:

desc: 'boolean, 1 if the animals was treated exactly once (not accounging for possible antibioprevention) and still alive, 0 if not treated or treated multiple times or dead'

treatment_successful:

desc: 'boolean value, 1 if the treatment will succeed, 0 otherwise'

# _____ _ _

# | __ \ | | | |

# | |__) | __ ___ | |_ ___ | |_ _ _ _ __ ___ ___

# | ___/ '__/ _ \| __/ _ \| __| | | | '_ \ / _ \/ __|

# | | | | | (_) | || (_) | |_| |_| | |_) | __/\__ \

# |_| |_| \___/ \__\___/ \__|\__, | .__/ \___||___/

# __/ | |

# |___/|_|

# SPECIFICATION OF INITIAL VARIABLE VALUES FOR SPECIFIC CASES

prototypes:

pen:

- default_pen:

desc: 'prototype to initialize pen variables'

pen_infection_duration: 0

pen_severe_clinical_duration: 0

pen_mild_clinical_duration: 0

pen_nb_dead_treated_distinct: 0

pen_nb_dead_infected_distinct: 0

pen_nb_dead_detected_distinct: 0

pen_nb_doses_dead: 0

pen_cumulate_incidence: 0

pen_prev_cumulate_incidence: 0

pen_previous_half_day_incidence: 0

pen_half_day_incidence: 0

pen_nb_MC_detections: 0

pen_nb_SC_detections: 0

pen_nb_MC_missed: 0

pen_nb_SC_missed: 0

pen_nb_false_positive: 0

pen_nb_true_negative: 0

pen_nb_deaths: 0

pen_time_first_detection: -1

pen_do_collective_treatment: 0

beef_calves:

#--------------------------------

# INITIALIZATION PROTOTYPES

#--------------------------------

- default_beef_calf:

desc: 'default prototype applied to all animals before any further modification'

treat_state: NT

clinical_state: A

detection_state: ND

nb_consecutive_doses: 0

treated: 0

treated_only_once: 0

infected: 0

detected: 0

evolve_to_death: 0

evolve_to_SC: 0

nb_doses_total: 'use_antibioprevention'

health_state: S

- initial_healthy_beef:

desc: 'prototype for healthy and susceptible animals'

health_state: S

- initial_carrier_beef:

desc: 'prototype for asymptomatic carriers'

health_state: pI

#--------------------------------

# MODIFICATION PROTOTYPES

#--------------------------------

- healthy:

desc: 'prototype for making individual return to S'

health_state: S

clinical_state: A

nb_consecutive_doses: 0

- undetected:

desc: 'restore the Undetected status'

detection_state: ND

- ignored:

desc: 'animal no more considered for further treatmens'

detection_state: Ig

- detected:

desc: 'mark an animal as detected'

detection_state: D

# _____ _ _ _ _

# |_ _| (_) | (_) | |

# | | _ __ _| |_ _ __ _| |

# | | | '_ \| | __| |/ _` | |

# _| |_| | | | | |_| | (_| | |

# |_____|_| |_|_|\__|_|\__,_|_|

# _ _ _ _

# | (_) | (_)

# ___ ___ _ __ __| |_| |_ _ ___ _ __ ___

# / __/ _ \| '_ \ / _` | | __| |/ _ \| '_ \/ __|

# | (_| (_) | | | | (_| | | |_| | (_) | | | \__ \

# \___\___/|_| |_|\__,_|_|\__|_|\___/|_| |_|___/

initial_conditions:

pen:

- prototype: initial_healthy_beef

amount: 'round(pen_size * (1 - initial_prevalence))'

- prototype: initial_carrier_beef

amount: 'round(pen_size * initial_prevalence)'

# ____ _ _

# / __ \ | | | |

# | | | |_ _| |_ _ __ _ _| |_ ___

# | | | | | | | __| '_ \| | | | __/ __|

# | |__| | |_| | |_| |_) | |_| | |_\__ \

# \____/ \__,_|\__| .__/ \__,_|\__|___/

# | |

# |_|

# TYPE AND PERIODICITY OF OUTPUTS

outputs:

type: csv

pen:

period: 1

extra_vars:

- total_pen

- pen_time_first_detection

- pen_nb_deaths

- pen_cumulate_incidence

- pen_prev_cumulate_incidence

- pen_half_day_incidence

- pen_previous_half_day_incidence

- pen_daily_incidence

- pen_nb_dead_treated_distinct

- pen_nb_treated_distinct

- pen_nb_treated_only_once

- pen_nb_infected_distinct

- pen_nb_detected_distinct

- pen_infection_duration

- pen_severe_clinical_duration

- pen_mild_clinical_duration

- pen_nb_doses

- pen_nb_MC_detections

- pen_nb_SC_detections

- pen_nb_MC_missed

- pen_nb_SC_missed

- pen_nb_false_positive

- pen_nb_true_negative

- pen_do_collective_treatment

...
